# Biological aspects of the tongue morphology of wild-captive WWCPS rats: a histological, histochemical and ultrastructural study

**DOI:** 10.1007/s12565-018-0445-y

**Published:** 2018-06-14

**Authors:** Karolina Goździewska-Harłajczuk, Joanna Klećkowska-Nawrot, Karolina Barszcz, Krzysztof Marycz, Tomasz Nawara, Klaudia Modlińska, Rafał Stryjek

**Affiliations:** 1 Department of Biostructure and Animal Physiology, Faculty of Veterinary Medicine, Wroclaw University of Environmental and Life Sciences, Norwida 25, 50-375 Wrocław, Poland; 2 Department of Morphological Sciences, Faculty of Veterinary Medicine, Warsaw University of Life Sciences, Warsaw, Poland; 3 Faculty of Biology, Electron Microscopy Laboratory, Wroclaw University of Environmental and Life Sciences, Wrocław, Poland; 4 Institute of Psychology, Polish Academy of Sciences, Warsaw, Poland

**Keywords:** Wild rat, Laboratory rat, WWCPS, Domestication, Tongue morphology

## Abstract

The aim of this study was to characterise the tongue in wild-type rats using several microscopic techniques. Warsaw Wild Captive Pisula Stryjek (WWCPS) rats belong to a lineage of wild-caught rats. The study was carried out on tongues of 15 male and 15 female WWCPS rats. Histological, histochemical and ultrastructural studies were carried out. There were no significant differences between the male and female WWCPS rat tongues. There was a median groove approximately 1 cm long in the apex of the tongue that faded caudally. The intermolar prominence was clearly marked in the distal part of the lingual body. Lingual mechanical papillae located on the surface of the tongue formed four subtypes based on their shape: small filiform papillae, giant filiform papillae, thin elongated filiform papillae and wide filiform papillae. Gustatory papillae formed the second group of papillae and were divided into bud-shaped fungiform papillae, a single vallate papilla surrounded by an incomplete papillary groove and foliate papillae, which were a well-formed and composed of several pairs of folds divided by longitudinal grooves. In the posterior lingual glands (mucoserous and serous), acidic sulphated mucin-secreting cells gave a strong AB pH 2.5 positive reaction, and a positive reaction with the AB pH 1.0 stain for acidic carboxylated mucin. Double AB/PAS staining showed the presence of the majority of mucous cells with predominant of acidic mucins. Positive PAS staining showed the presence of neutral mucin. HDI staining demonstrated a weak positive reaction within Weber’s glands of the WWCPS rat tongue.

## Introduction

WWCPS (Warsaw Wild Captive Pisula Stryjek) rats belong to a lineage of wild-caught brown rats (*Rattus norvegicus*), which was recognised in 2007 and is registered at the Polish Patent Office under number Z-320033 (Stryjek and Pisula 2008). Currently, these animals are used in numerous physiological, behavioural, neurological and pharmacological studies (Stryjek et al. 2012a, 2012b, 2013). These rats have several different behavioural characteristics compared to laboratory rats, and albino and pigmented strains of laboratory rats (Stryjek et al. 2012b, 2013; Himmler et al. 2013; Modlińska and Stryjek 2016). Wild rat domestication based on multigenerational breeding led to several phenotypical changes, while other features have been acquired as a result of adaptation to living conditions (also through epigenetic changes) (Jensen 2010). This has caused morphological changes and a regression of selected sensory organs (Lockard 1968; Stryjek and Modlińska 2016). The differences between the lines are further increased by the fact that many laboratory rats are albino animals. Albinism, which is triggered by a mutation of a single gene occurring once in >12,000 births, results in lack of secretion of the tyrosinase enzyme, which is responsible for melanin production. Albinism is inherited—it is conditioned by a recessive gene (Grønskov et al. 2007). Achromasia is a trait that makes adaptation more difficult (in natural conditions), as the albino animal stands out of the group due to its colour, which makes it an easy prey for predators (Dröscher 1993). In albino strains of rats, the retina also lacks pigmentation. The retina becomes translucent, which leads to visual impairment (Prusky et al. 2002), this means that the albino individuals have a weaker sense of vision than pigmented individuals. In addition to the visual impairment, the albino rats have an underdeveloped sense of smell compared with their pigmented counterparts. Albino rats exhibit weaker responses to the odour of female rats in oestrus (Sachs 1996). On the other hand, a study by Shumake et al. (1971) found that domestication has not changed taste preferences in rats. Wild rats (unchanged by domestication) have features formed by the natural environment. Hence, comparative studies of wild and domesticated lines are valuable.

The tongue is an important organ enabling food intake and the sensation of taste (Iwasaki 2002). Due to its characteristic structure, it takes part in food grinding as well as moving the food toward the throat (Doran and Baggett 1971). Papillae that are present on the surface of the tongue are divided, based on their function, into mechanical papillae and gustatory papillae. Depending on the region of the tongue, it is covered by a keratinised stratified squamous epithelium or a non-keratinised stratified squamous epithelium. The keratinized stratified squamous epithelium is divided into several layers: the stratum basale (*stratum basale*), the stratum spinosum (*stratum spinosum*), the stratum granulosum (*stratum granulosum*) and the stratum corneum (*stratum corneum*) (Iwasaki et al. 1999; Ciena et al. 2013; Watanabe et al. 2013). There are significant differences both in the degree and the time of keratinisation (Iwasaki et al. 1999). The thickest layer of keratin covers the mechanical papillae, which primarily form a protective layer on the surface of the tongue. Ultrastructural studies assessing the relationship between the formation of filiform papillae and keratinisation of the lingual epithelium in Sprague-Dawley rats showed that morphogenesis of the lingual filiform papillae depends on the degree of keratinisation of the epithelium on the surface of the tongue immediately before birth and several weeks after birth (Iwasaki et al. 1999). This is associated with the environmental changes of the foetus and newborn animal (Iwasaki et al. 1999). Transmission electron microscopy (TEM) showed that keratohyaline granules are present in newborn Sprague-Dawley rats (Iwasaki et al. 1999).

Muscle tissue with variously arranged skeletal muscle fibers (longitudinal, transverse and vertical muscle fibers as *musculus lingualis proprius*) comprises the majority of the mammalian tongue (Doran and Baggett 1971). Within this tissue there are lingual glands, which are located in the rostral part of the tongue (glands of Blandin-Nuhn) and glands within the caudal part of the tongue (von Ebner’s glands and Weber’s glands) (Tandler et al. 1994; Nagato et al. 1997). Based on previous studies, the lingual glands can be divided into apical glands, glands accompanying the vallate and foliate papillae and glands of the root of the tongue (Nagato et al. 1997).

The structure of the rat tongue in different strains and breeds of rats has been described previously using light microscopy and ultrastructural studies (Hosley and Oakley 1987; Iwasaki et al. 1987, 1997, 1999; Kullaa-Mikkonen et al. 1987; Nagato et al. 1997; Iino and Kobayashi 1988; Jakob et al. 1998; Wakisaka et al. 1998; Triantafyllou et al. 2002; Yücel et al. 2002; de Abreu et al. 2006; Picoli et al. 2006; Verli et al. 2008; Abayomi et al. 2009; Lopes et al. 2009; Costa et al. 2013; Al-Refai et al. 2014; El Sharaby et al. 2014; Reginato et al. 2014). However, the majority of those studies did not assess gender differences, and differences between wild and laboratory rats. Numerous histological and histochemical studies, as well as scanning electron microscopy (SEM) and TEM, have enabled the identification of significant differences in the microstructure of the lingual surface in other selected rodents of the *Rodentia* order (Kobayashi 1990; Kobayashi et al. 1992; Grandi et al. 1994; Stangl and Pfau 1994; Whitehead and Kachele 1994; Watanabe et al. 1997, 2013; Emura et al. 1999a, b, 2001, 2011; Jackowiak and Godynicki 2005; Ünsaldi 2010; Shindo et al. 2006; Kulawik and Godynicki 2007; Toprak and Yilmaz 2007; Nonaka et al. 2008; Cheng et al. 2009; Kilinc et al. 2010; Alvarez et al. 2011; Atalar and Karan 2011; Karan et al. 2011; Abumandour and El-Bakary 2013; Ciena et al. 2013, 2017; Sakr et al. 2013; Wołczuk 2014; Cizek et al. 2016; Sadeghinezhad et al. 2018; Wannaprasert 2018).

The aim of this study was to characterise the microstructure of the tongue and lingual glands in wild undomesticated WWCPS rats with the use of current microscopic imaging techniques, including light microscopy, SEM and TEM. The second aim was to compare the obtained results with those obtained by other authors in studies carried out on laboratory rats and other Rodentia.

## Materials and methods

### Animals

The study was carried out on 6-month-old WWCPS rats. All the animals were clinically healthy, kept in standard living conditions and fed a uniform diet. All rats were housed in groups of three to five in Eurostandard type IV cages with ad libitum access to water and standard laboratory fodder (Labofeed H, WP Morawski, Kcynia, Poland). The day/night cycle was set at 12 h/12 h. The study material was obtained from the Institute of Psychology of the Polish Academy of Sciences in Warsaw. The permission to breed laboratory animals by the Institute of Psychology of the Polish Academy of Sciences in Warsaw was granted by the District Veterinary Officer in Warsaw (identification no. 146571035, decision no. 167/2013 from 30.12.2013). Morphological studies of the rat tongues were carried out post mortem at the Department of Animal Anatomy of Wroclaw University of Environmental and Life Sciences. The examination of tissues obtained post mortem can be done without approval of the Ethics Committee (Official Journal of the European Union L276/33: Directive of the European Parliament and Council 2010/63/UE; Journal of Laws of the Republic of Poland, Item 266, Act 15 January 2015). The WWCPS rats were divided into two groups. The first group consisted of males (*N* = 15) and the second was females (*N*  = 15). Tongues were collected from all the animals in both groups (*N* = 30). They were assessed macroscopically and measured. The length and width of the tongues were evaluated at the apex, body and root of the tongue. Macroscopic photographs of the animals and the tongues were taken using a Canon 400EOS camera and a stereoscopic Zeiss Stemi 2000-C microscope (Zeiss, Jena, Germany). Macroscopic measurements were carried out using an electronic slide caliper with an accuracy of 0.1 mm. The results of the macroscopic study underwent analysis, and the mean ± standard deviation was calculated using statistical software (Microsoft Office Professional Plus, 2013, Microsoft, Redmont, WA). This study was supported by the Wroclaw Centre of Biotechnology, the Leading National Research Centre (KNOW) for 2014–2018 (Agreement No. 7/PB/2015/KNOW).

### Histological and histochemical studies

Six randomly chosen tongues from the 15 tongues collected from males, and 6 randomly chosen female tongues from a total of 15 tongues were fixed in 4% formaldehyde solution, buffered (Chempur, Poland), and subjected to histological and histochemical analysis.

### Histological study

Samples were collected from the apex, body and root of the tongue together with the lingual glands. Next, they were washed in running water for 24 h. The samples were then processed in a vacuum tissue processor—ETP (RVG3, INTELSINT, Italy), embedded in paraffin and cut using a Slide 2003 (Pfm, Cologne, Germany) sliding microtome into 3–4 µm sections. The hematoxylin and eosin (H&E) (general structure), Masson–Goldner trichrome (connective tissue, keratin layer), and Azan trichrome (connective tissue, collagen fibers) staining methods were used in order to assess the proper histological structure of the assessed tissue.

### Histochemical study

Histochemical analysis of the lingual glands was carried out using the periodic acid-Schiff method (PAS) in order to identify glycans, glycoconjugates and neutral or weakly acidic glycoprotein (Sheehan and Hrapchak 1980). Moreover, the alcian blue pH 2.5 (AB pH 2.5) method was used to identify sulphated and carboxylated acid mucopolysaccharides and sulphated and carboxylated sialomucins, while, additionally, AB pH 1.0, detected the strongly sulphated mucosubstances (Schumacher et al. 2004; Sadeghinezhad et al. 2018). Hale’s dialysed iron staining (HDI) method was used to identify sulphated mucosubstances (SAM) and carboxylated mucosubstances (CAM) (Munakata et al. 1985). AB with the pH 2.5/PAS staining was used to detect acidic and neutral mucin. The samples were examined using the Zeiss Axio Scope A1 light microscope (Zeiss, Jena, Germany) and assessed according to the method described by Spicer and Henson (1967).

### Scanning electron microscopy

Samples from six male and six female tongues (others tongues than for histological study) were collected. For ultrastructural studies, the tongues were fixed in 2.0% glutaraldehyde dissolved in 0.1 M phosphate buffer at pH 7.4. The research material was rinsed in a phosphate buffer. After being fixed, the samples were dehydrated in up to 50% ethanol and stored at room temperature for 2 h. The process was repeated with 60%, 70, 80, 90, 98, 100% ethanol. Dehydrated tissue was dried (critical point). The samples were coated with gold using ScanCoat (Edwards) and viewed using a SE1 detector at a 10 kV filament tension (SEM, Zeiss Evo LS 15). SEM analysis was carried out at the Laboratory of Electron Microscopy of the Wroclaw University of Environmental and Life Sciences.

### Transmission electron microscopy

Specimens for the TEM study were collected from the tongues. The samples were fixed in 2.0% glutaraldehyde dissolved in pH 7.4 0.1 M phosphate buffer for 2 weeks and were rinsed in a phosphate buffer. The material was then post-fixed in 4% OsO4 (osmium tetroxide) for 2 h at room temperature. Following rinsing of the samples in a phosphate buffer, they were dehydrated in an acetone series (30–100%). Then, the research material was immersed in an Epon 812 epoxide resin. The samples were cut into 70 nm sections with a diamond knife using a Leica Reichert Ultracut E microtome (Leica Microsystems, WetzlarGermany) (Kubrakiewicz et al. 2003). The sections were assessed using the Tesla BS 540 transmission electron microscope. The TEM studies were carried out at the Electron Microscopy Laboratory of the Wroclaw University.

## Results

### Gross morphology of the tongue

**Fig. 1:**
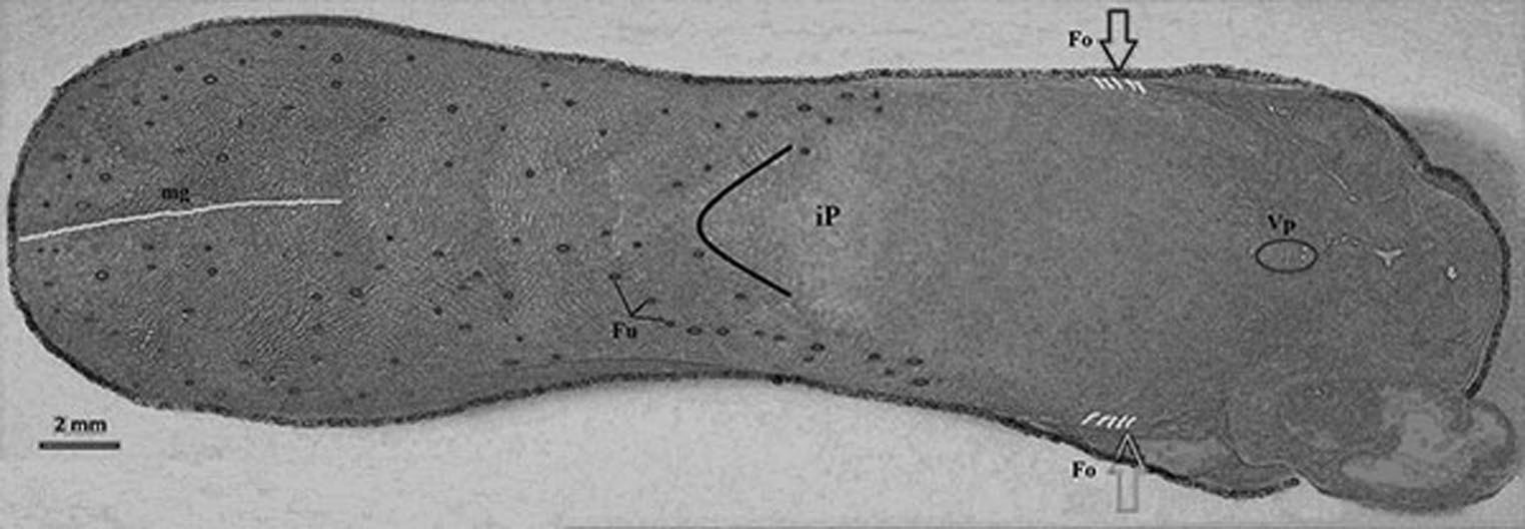
Schematic diagram of the Warsaw wild captive Pisula Stryjek (WWCPS) rat tongue. *Fo* Foliate papilla, *Fu* fungiform papilla, *iP* intermolar prominence, *mg* median groove, *Vp* vallate papilla

**Fig. 2a–i:**
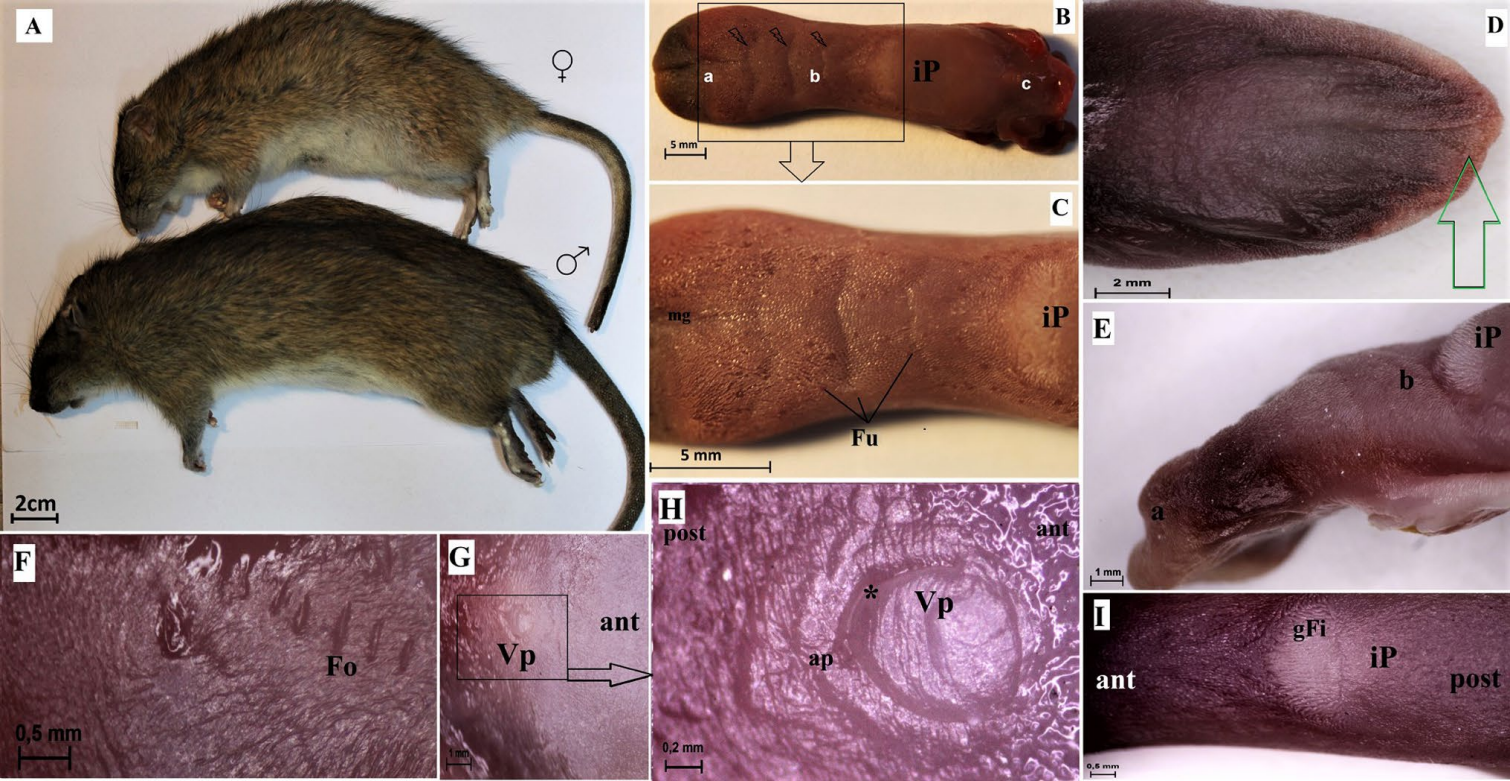
Macroscopic characterization of WWCPS rats and their tongue. **a** A male and female WWCPS rat. **b** Macroscopic view of the WWCPS rat tongue:* a* apex,* b* body,* c* root. **c** Distribution of the fungiform papillae on the dorsal surface of the lingual apex and body with a clearly visible median groove within the lingual apex. **d** Ventral smooth surface of the lingual apex. **e** A lateral position of the lingual apex and body with a well visible intermolar prominence. **f** Area of the foliate papillae. **g** Area of the vallate papilla between body and root of the tongue. **h** Magnification of the vallate papilla with the annular pad. **i** Visible giant filiform papillae within the intermolar prominence. *ant* anterior, *ap* annular pad, *Fo* foliate papilla, *Fu* fungiform papilla, *gFi* giant filiform papillae, *iP* intermolar prominence, *mg* median groove, *post* posterior, *Vp* vallate papilla.* Bars*** a** 2 cm;** b**,** c** 5 mm;** d** 2 mm;** e**,** g** 1 mm;** f**,** i** 0.5 mm;** h** 0.2 mm

**Fig. 3:**
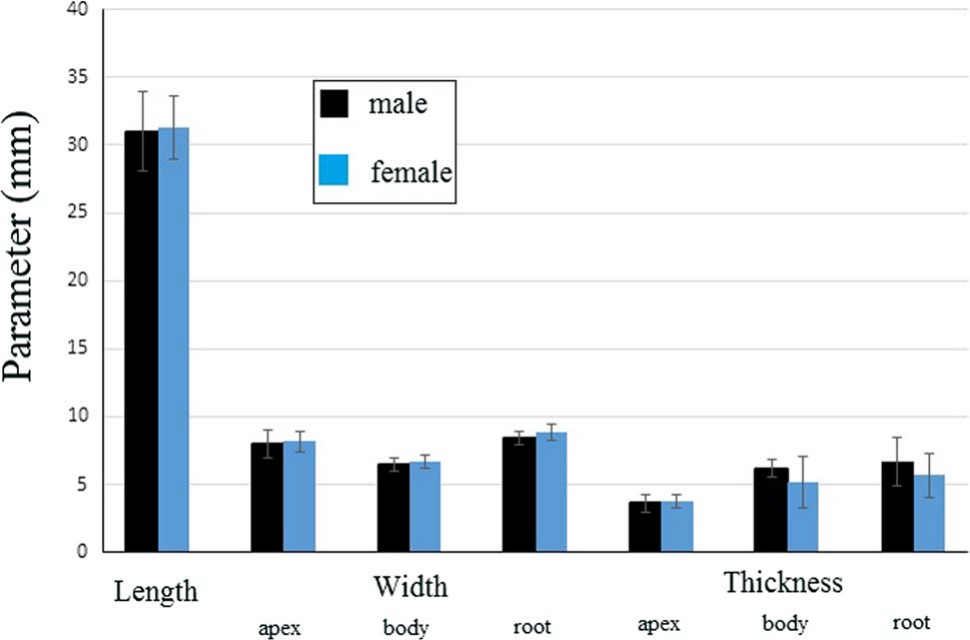
Morphometric analysis of the tongue (length, width and thickness; mm ± SD) in male and female WWCPS rats

The wild-type rat tongue consists of three main parts: the apex, the body and the root. The apex was smooth and rounded (Figs. 1, 2). Macroscopically, part of the body of the tongue was narrower than the apex (Fig. 1, 2). The measurements of selected parts of the tongue based on gender are presented in Fig. 3. No statistically significant differences in the measurements were found between males and females (Fig. 3). There was a median groove approximately 1 cm long in the apex of the tongue that faded caudally toward the body of the tongue. Moreover, an intermolar prominence was present in the lingual body (Fig. 2b, c, e, i). There were three transverse depressions on the surface of the lingual body, which were post-mortem impressions of the palatal rugae (Fig. 2b, c). Filiform papillae and three types of gustatory papillae were clearly visible on the surface of the tongue: fungiform papillae, vallate papilla and foliate papillae (Fig. 2). Within the intermolar prominence, the filiform papillae were directed rostrally and laterally (Fig. 2c, e, i). The fungiform papillae were distributed in the apex as well as the body of the tongue outside the areas filled with giant filiform papillae (Fig. 2b, c). The single vallate papilla was localised in the midline of the tongue between its body and root (Fig. 2g, h). The annular pad surrounding the vallate papilla was not marked clearly (Fig. 2g, h). The foliate papillae were localised posterolaterally between the body and the root of the tongue, and were usually formed from five even epithelial folds (Fig. 2f). Small filiform papillae and a few fungiform papillae were localised on the ventral surface of the tongue around the edges of the apex, and the remaining surface of the ventral surface was smooth (Fig. 2d, e).

### Histological and ultrastructural study: SEM and TEM

#### Filiform papillae

**Fig. 4a–j:**
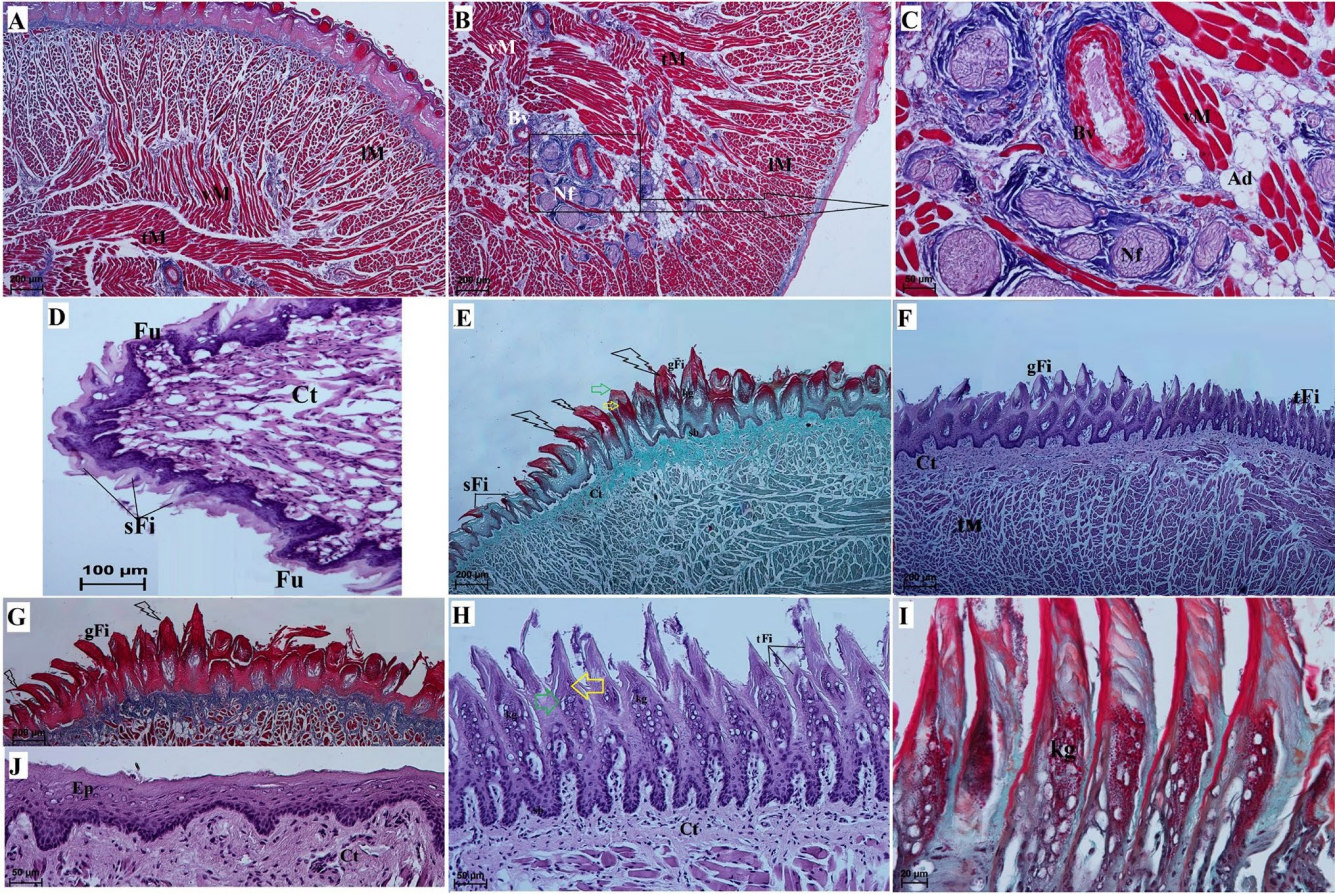
Characterisation of the structure of the filiform papillae in the WWCPS rat tongue using light microscopy. **a** Transverse cross section of the tongue, dorsal and lateral part of tongue. Azan staining. **b** Lateral and ventral part of tongue. Azan staining. **c** magnification of nerve fibres. Azan staining. **d** apex of the tongue. H&E staining. **e** Transverse cross section of the anterior part of the intermolar prominence. Note clearly visible keratin layer on the giant filiform papillae surface,*arrows* keratin spines,* yellow arrow* anterior part of the filiform papillary processes,* green arrow* posterior part of the filiform papillary processes. Masson-Goldner trichrome staining. **f** Longitudinal cross section of the giant filiform papillae and the thin elongated filiform papillae. H&E staining. **g** A transverse cross section of the anterior part of the intermolar prominence showing keratin spines on the apex of the giant filiform papillae. Azan trichrome staining. **h** A longitudinal cross section of the thin elongate filiform papillae. H&E staining. **i** Magnification of the thin elongate filiform papillae with well visible keratohyaline granules. Masson-Goldner staining. **j** Ventral surface of the tongue without papillae. H&E staining. *Ad* adipose tissue, *Ct* connective tissue, *Ep* epithelium, *gFi* giant filiform papillae, *kg* keratohyaline granules, *lM* longitudinal muscle fibres, *Nf* nerve fibres, *sb stratum basale*, *sFi* small filiform papillae, *tFi* thin elongate filiform papillae, *tM* transverse muscle fibres, *vM* vertical muscle fibres.* Bars* **a**, **e**–**g** 200 µm;** b**, **c**, ** h**,** j** 50 µm;** d** 100 µm;** i** 20 µm

**Fig. 5a–l:**
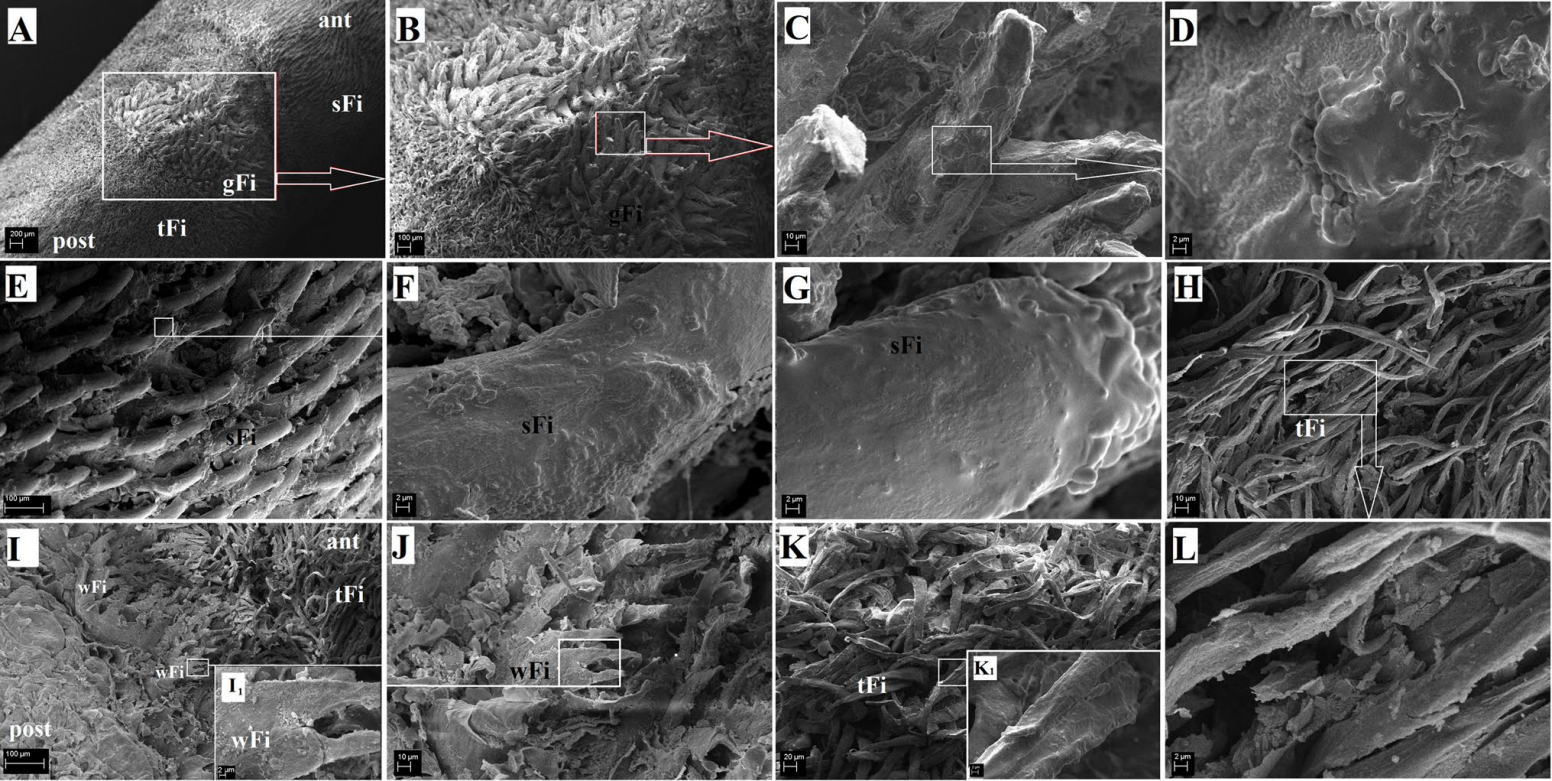
Microstructure of the four different types of the lingual filiform papillae on the WWCPS rat tongue surface in scanning electron microscopy (SEM). **a** Visualization of the shape of the filiform papillae: small filiform papillae on the body of the tongue, within the intermolar prominence the giant filiform papillae as wheel as thin filiform papillae. **b** Giant filiform papillae with various positions of the papillary apex. **c** Magnification of the giant filiform papillae. **d** Irregular surface of the giant filiform papilla. **e** Small filiform papillae with a rounded tip within the lingual body. **f** Magnification of the small filiform papilla. **g** Irregular surface of the small filiform papilla. **h** Thin elongated filiform papillae located caudally to the giant filiform papillae. **i** Width of the filiform papillae located between the lingual body and root of the tongue. Magnification of the width of the filiform papillae. **j** Several widths of the filiform papillae. **k** Shape of the thin elongated filiform papillae. **l** Magnification of a single thin elongated filiform papilla.* ant* Anterior, *gFi* giant filiform papillae,* post* posterior, *sFi* small filiform papillae, *tFi* thin elongate filiform papillae, *wFi* width filiform papillae.* Bars* **a** 200 µm; **b**, **e**, **i** 100 µm; **c**, **h**, **j** 10 µm; **d**–**g**, **i**_**1**_, **l** 2 µm; **k**, **k**_**1**_ 20 µm

The filiform papillae were the most common type of lingual papillae located on the surface of the WWCPS rat tongue. Their shape varied depending on the region of the tongue (Figs. 4, 5). In the apex and the cranial 2/3 of the body of the tongue, there were small filiform papillae with a single conical pointed tip (Figs. 2c, 5a). In the apex, the small filiform papillae were shorter than those in the body of the tongue. Exfoliated epithelial cells were visible on the small filiform papillae. In the distal part of the body, giant filiform papillae with a pointed or bifurcated tip but without additional projections at the base of the papillae were located directly on the intermolar prominence (Fig. 5a, b). In the SEM study, the giant filiform papillae were directed caudally, laterally and rostrally (Fig. 5a, b). The surface of the giant filiform papillae was irregular and single exfoliated epithelial cells were visible (Fig. 5c, d). The most caudal surface of the intermolar prominence contained elongated papillae known as thin elongated filiform papillae (Fig. 5h, i, k, k_1_, l). Those thin elongated filiform papillae were much thinner than the small filiform papillae and were directed towards the root of the tongue (Fig. 5a, b). Some of the thin elongated filiform papillae contained small pseudopapillae on their surface (Fig. 5k_1_). In addition, filiform papillae known as wide filiform papillae were located laterally immediately behind the thin elongated filiform papillae in the root of the tongue and contained four to five additional projections located at their tips (Fig. 5i, i_1_). Each filiform papilla had an anterior and posterior surface (Figs. 4, 5). Histological examination of the filiform papillae revealed the presence of numerous keratohyaline granules on the surface of the anterior processes of the filiform papillae. These keratohyaline granules were not visible on the epithelial surface of the posterior processes of the filiform papillae (Fig. 4e–h). The giant filiform papillae were covered by a thick keratin layer (Fig. 4e, h). In that layer, the cell nuclei faded superficially forming an orthokeratinised epithelium (Fig. 4e, h). A thin layer of connective tissue with numerous connective tissue fibres, fibroblasts and blood vessels was present under the layer of filiform papillae (Fig. 4e–g). The cells of the filiform papillary *stratum basale* were cuboidal, while the nuclei of the *stratum basale* were oval (Fig. 4g). The small filiform papillae were 283.09 ± 45.51 µm long on the apex and the body of the tongue. The giant filiform papillae were 467.81 ± 72.18 µm long. The thin elongated filiform papillae were 324.24 ± 38.05 µm long. The small filiform papillae were 128.31 ± 22.3 µm wide on the apex and the body of the tongue. The giant filiform papillae were 146.57 ± 17.44 µm wide. The thin elongated filiform papillae were 73 ± 8.93 µm wide. The ventral surface of the apex of tongue was covered by a thin keratin layer, while the ventral surface of the body of the tongue was smooth without papillae (Fig. 4j). On the ventral surface of the tongue, the epithelial cells of the *stratum basale* were cuboidal and the nuclei were oval (Fig. 4j).

#### Fungiform papillae

**Fig. 6:**
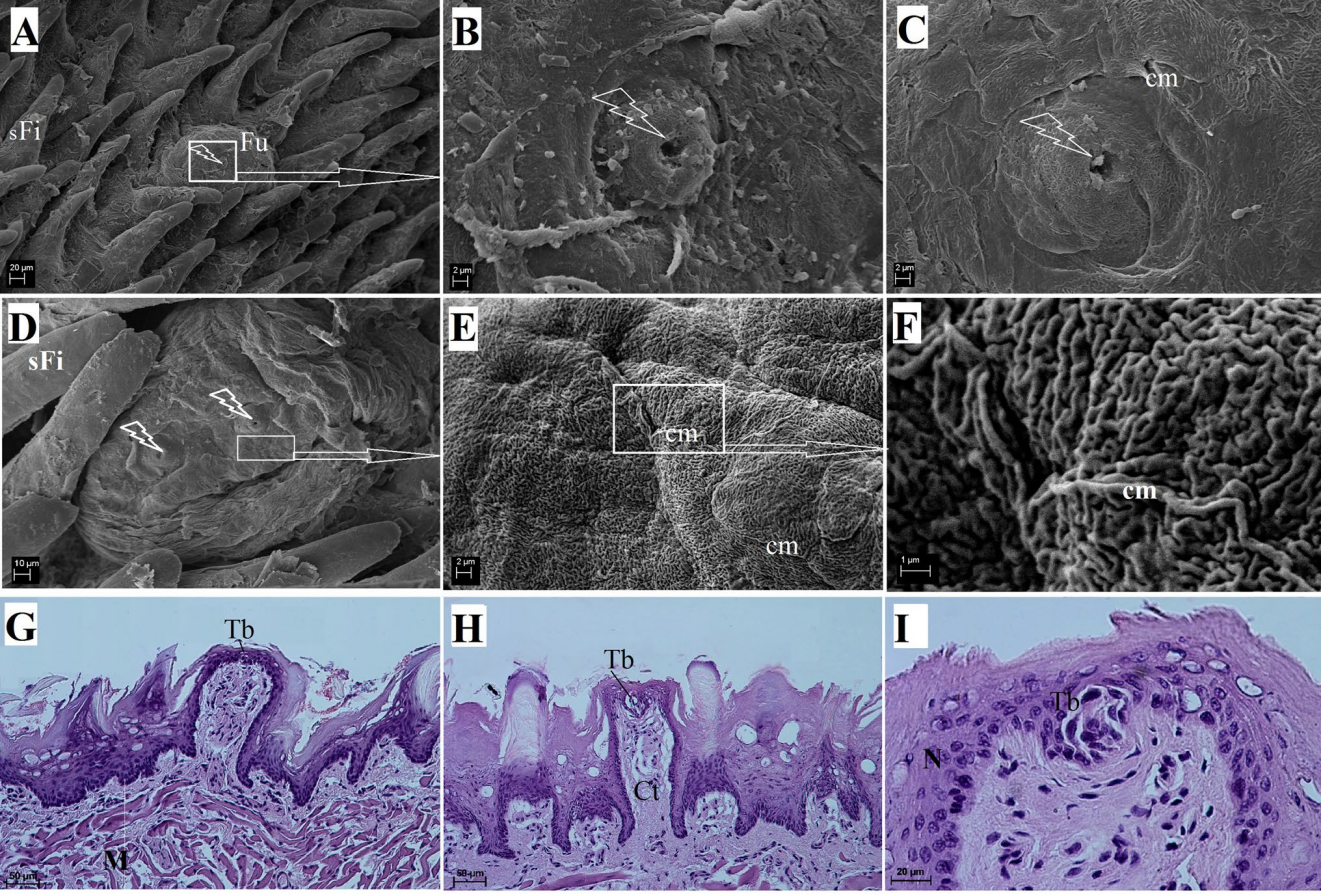
The surface of the fungiform papilla of the tongue of the WWCPS rat in SEM (**a**–**f**) and the longitudinal cross section of the fungiform papilla in light microscopy (**g**–**i**). **a** Rounded fungiform papilla with taste pore.* Arrow* Papilla located between filiform papillae within the lingual body. **b** Structure of the taste pore. **c** Magnification of the taste pore with visible epithelial cells which were connected in cell margins. **d** Fungiform papilla with double taste pores. **e** Irregular surface of the fungiform papilla with microfolds. **f** Magnification of microfolds. **g** Rounded fungiform papilla without a keratin layer with a visible taste bud. H&E staining. **h** H&E staining. **i** Magnification of the single taste bud of the fungiform papilla. H&E staining. *cm* Cell margins, *Ct* connective tissue, *Fu* fungiform papilla, *M* muscles, *N* nucleus, *sFi* small filiform papillae, *Tb* taste bud.* Bars* **a** 20 µm; **b**, **c**, **e** 2 µm; **d**10 µm; **f** 1 µm; **g**, **h** 50 µm; **i** 20 µm

The fungiform papillae were distributed equally in the apex and the body of the tongue excluding the areas covered by giant filiform papillae (Fig. 2b). The fungiform papillae were round (Fig. 6a, d) and there were taste pores visible on their surface (Fig. 6a–d). The SEM study revealed that the surface of the epithelial cells of the fungiform papillae consisted of microfolds and micropits (Fig. 6e, f). The fungiform papillae were 220.17 ± 22.5 µm long and 145.96 ± 23.26 µm wide. The histological examination of the surface of the fungiform papillae revealed that they were covered by a thin keratin layer (Fig. 6g, i). The taste buds had a barrel-shape in a transverse cross section (Fig. 6i).

#### Vallate papilla

**Fig. 7:**
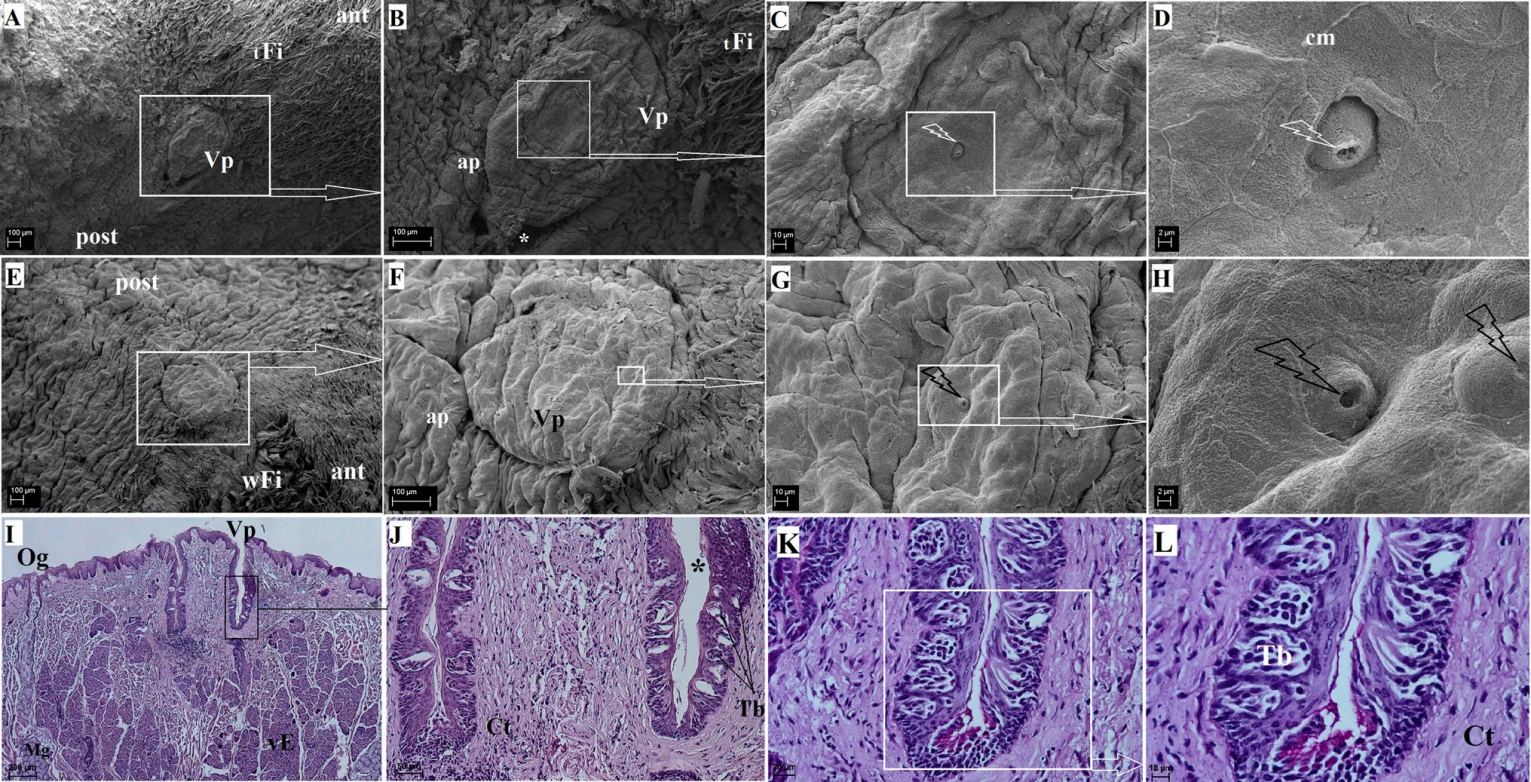
The surface of the vallate papilla of the tongue of the WWCPS rat in SEM (**a**–**h**) and the cross section of the vallate papilla area in light microscopy (**i**–**l**). **a** Single oval (drop-like) vallate papilla located between the lingual body and root. **b** Magnification of the vallate papilla with a clear papillary groove (*asterisk*), and annular pad. **c** Irregular surface of the vallate papilla with a centrally located taste pore (*arrow*). **d** Cell margins between consecutive epithelial cells with visible microfolds and micropits. **e** Round vallate papilla. **i** Cross section of the vallate papillae with a papillary groove, von Ebner’s gland is present beneath this papilla. H&E staining. **j** Shape of numerous taste buds of the vallate papillae. H&E staining. **k** Magnification of the taste buds. PAS staining. **l** PAS staining. *ant* anterior, *ap* annular pad, *cm* cell margins, *Ct* connective tissue core, *mG* muco-serous glands, *Og* opening of lingual glands, *post* posterior, *Tb* taste buds, *tFi* thin elongate filiform papillae, *vE* von Ebner’s glands, *Vp* vallate papilla.* Bars* **a**, **b**, **e**, **f** 100 µm; **c**, **g**, **l** 10 µm; **d, h** 2 µm; **i** 200 µm; **j** 20 µm; **k** 50 µm

A single vallate papilla was located on the border between the body and the root of the tongue (Fig. 2g, h). It was located centrally (Figs. 2g, h, 7a, b, e, f) and had either an oval, laterally flattened shape or was round in some individuals (Fig. 7a, b, e, f). It was surrounded by a poorly marked annular pad and a papillary groove present at the border between the papilla and the pad (Fig. 7b, d). On the anterior part of the annular pad the width filiform papillae were present (Fig. 7b). Although the surface of the vallate papilla was irregular, it did not contain additional pseudopapillae (Fig. 7b, d). Studies using SEM revealed that taste bud pores were present on the surface of the vallate papilla (Fig. 7c, d, g, h) and ranged from one to several depending on the individual. The surface of the vallate papilla was covered by marginal cells that contained numerous microfolds and micropits (Fig. 7d, h). The vallate papilla was 711.12 ± 58.5 µm tall and 445.36 ± 29.11 µm wide. The histological examination revealed that the surface of the vallate papilla was covered by a very thin keratin layer (Fig. 7i). In addition, the connective tissue core contained numerous papillary projections in the transverse section (Fig. 7i). The taste buds were located in the wall of the annulary pad as well as in the lateral walls of the vallate papilla itself (Fig. 7i, j). They were barrel-shaped and contained receptor cells (Fig. 7k, l). Numerous serous glands (von Ebner’s glands) were located underneath the vallate papilla (Fig. 7). The excretory duct was directed toward the papillary groove (Fig. 7).

#### Foliate papillae

**Fig. 8:**
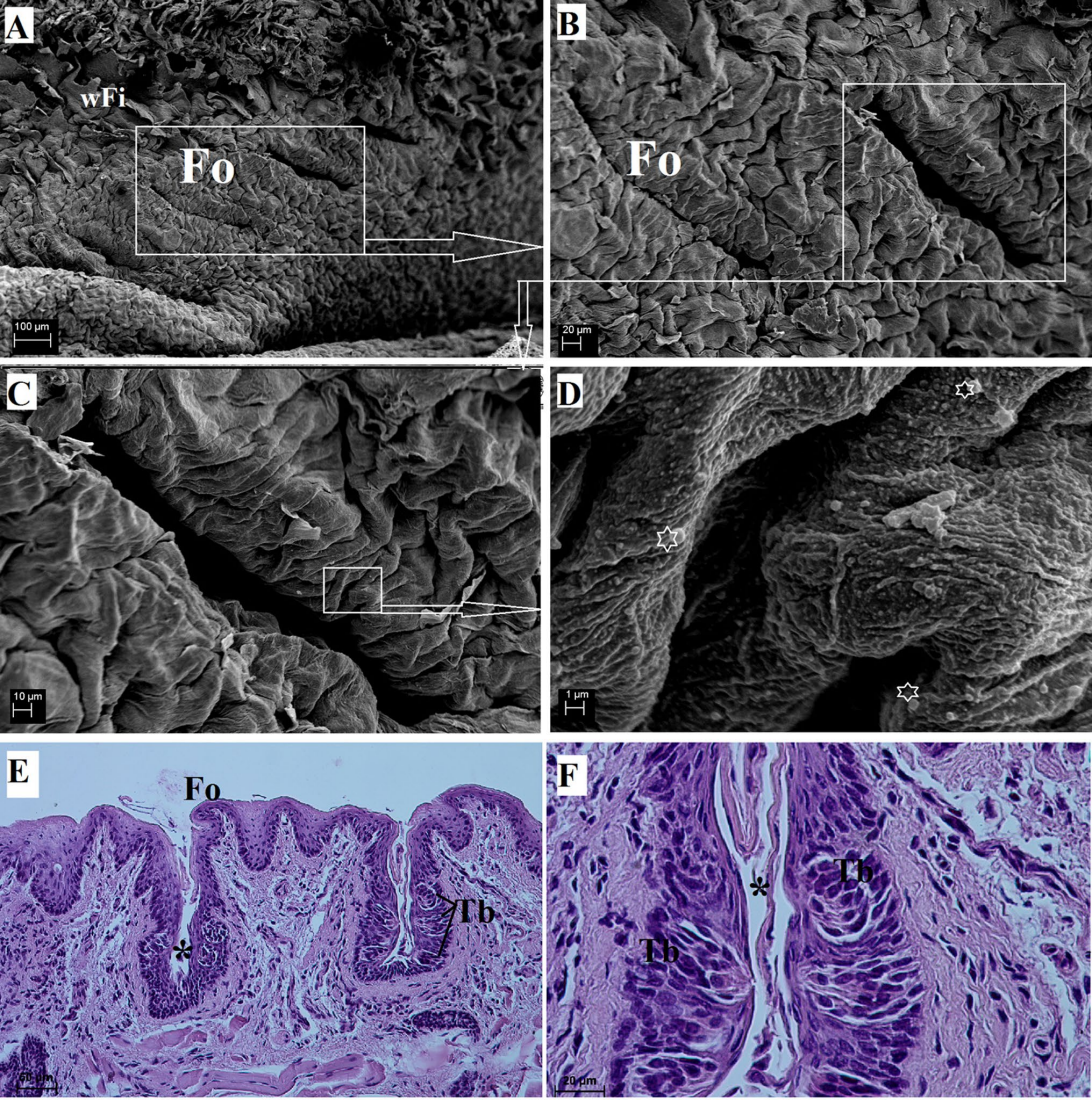
Surface of the foliate papillae of the tongue of the WWCPS rat in SEM (**a**–**d**) and the longitudinal cross section of the area of the foliate papilla in light microscopy (**e**, **f**). **a** Several lines forming the area of the foliate papillae. **b**, **c** Magnification of the foliate papillae. **d** Microfolds and micropits on the surface of the foliate papillae. **e** Shape of the foliate papilla in cross section. H&E staining. **f** Elongated taste buds of the foliate papilla with a foliate papillary groove (*asterisk*). H&E staining. *Fo* Foliate papilla, *Tb* taste buds, *wFi* width filiform papillae.* Bars* **a** 100 µm; **b**, **f** 20 µm; **c** 10 µm; **d** 1 µm; **e** 50 µm

The foliate papillae were well-developed in the WWCPS rats (Fig. 2f). They were located on the sides of the tongue at the border of the body and the root of the tongue. There were usually five pairs of epithelial folds (ridges) on the surface of these papillae, and they were separated by deep parallel grooves (Figs. 2f, 8a, b). The surface of the foliate papillae was covered by numerous microfolds and micropits (Fig. 8d). Single bacteria were visible on the surface of foliate papillae (Fig. 8d). The foliate papillae were 291.67 ± 98.6 µm long and 218.27 ± 48.91 µm wide. The histological study of the foliate papillae revealed the presence of taste buds in the walls of their individual folds (Fig. 8e). The taste buds were elongated or barrel-shaped (Fig. 8f). There was a thin layer of connective tissue under the papillae, which covered a layer of striated muscle and serous glands (Fig. 8e).

#### Lingual glands

**Fig. 9a–f:**
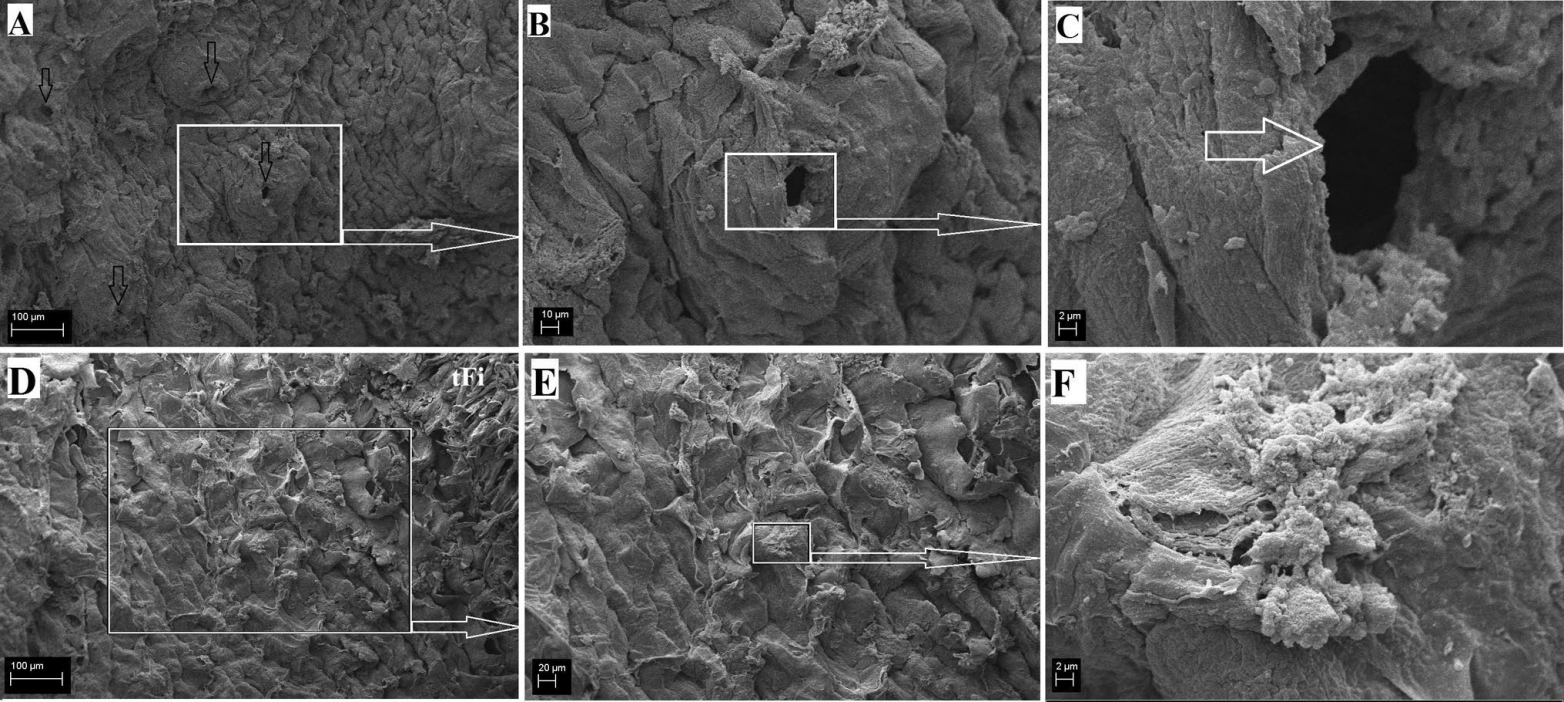
The surface of the root of the WWCPS rat tongue in SEM. **a** Openings of the lingual glands (*arrows*). **b** Magnification of the opening of the lingual glands. **c** Irregular surface of the lingual root. **d**, **e** Magnification of the irregular surface of lingual root. **f** Connection between consecutive cells on the root surface. *tFi* Thin elongate filiform papillae.* Bars* **a** 100 µm; **b** 10 µm; **c**, **f** 2 µm; **d** 100 µm; **e** 20 µm

**Fig. 10:**
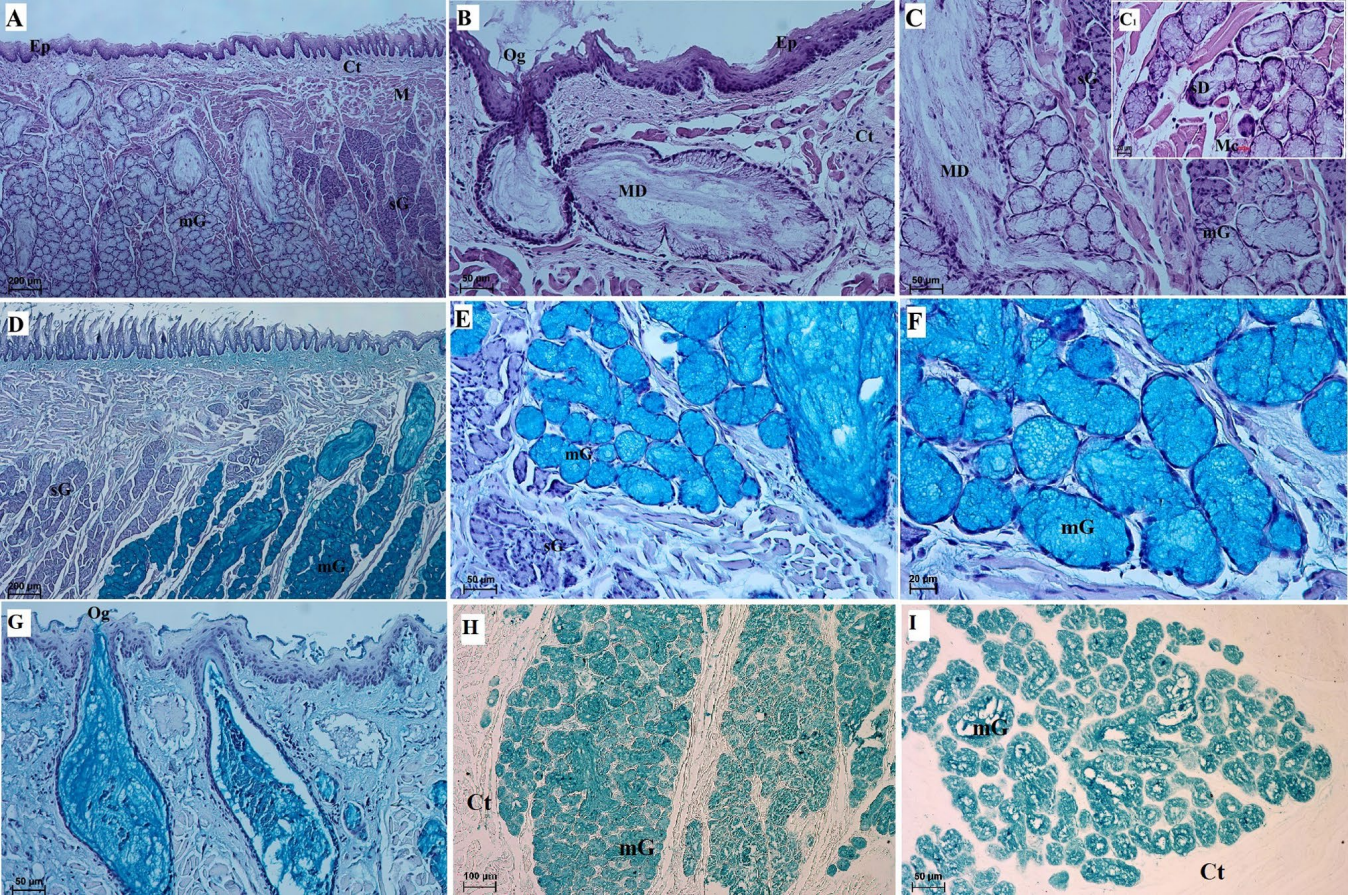
Histochemical analysis of the posterior lingual glands of the tongue of the WWCPS rat (von Ebner’s glands and Weber’s glands). **a** General structure of the lingual glands. H&E staining. **b** Opening of Weber’s lingual gland with main duct. H&E staining. **c** H&E staining. **c**_**1**_ Magnification of the mucoserous glands with well signed serous demilunes. H&E staining. **d** Serous glands and mucoserous glands. Alcian blue pH 2.5 staining. **e** Strong positive reaction (+++) in the mucous cells of mucoserous glands and a negative reaction in the serous glands. Alcian blue pH 2.5 staining. **f** Strong positive reaction (+++) in the mucoserous glands. Alcian blue pH 2.5 staining. **g** Two openings of Weber’s glands. Alcian blue pH 2.5 staining. **h** Weak positive reaction (+) in the mucous cells of mucoserous glands. Alcian blue pH 1.0 staining **i** Magnification of mucoserous glands. Alcian blue pH 1.0 staining. *Ct* Connective tissue, *Ep* epithelium, *MD* main duct, *mG* mucoserous glands, *M* muscles, *Mc* myoepithelial cells, *Og* opening of the lingual glands, *sD* serous demilunes, *sG* serous glands.* Bars* **a**, **d** 200 µm; **b**, **c**, **e**, **g**, **i** 50 µm; **c**_**1**_, **f** 20 µm; **h** 100 µm

**Fig. 11a–i:**
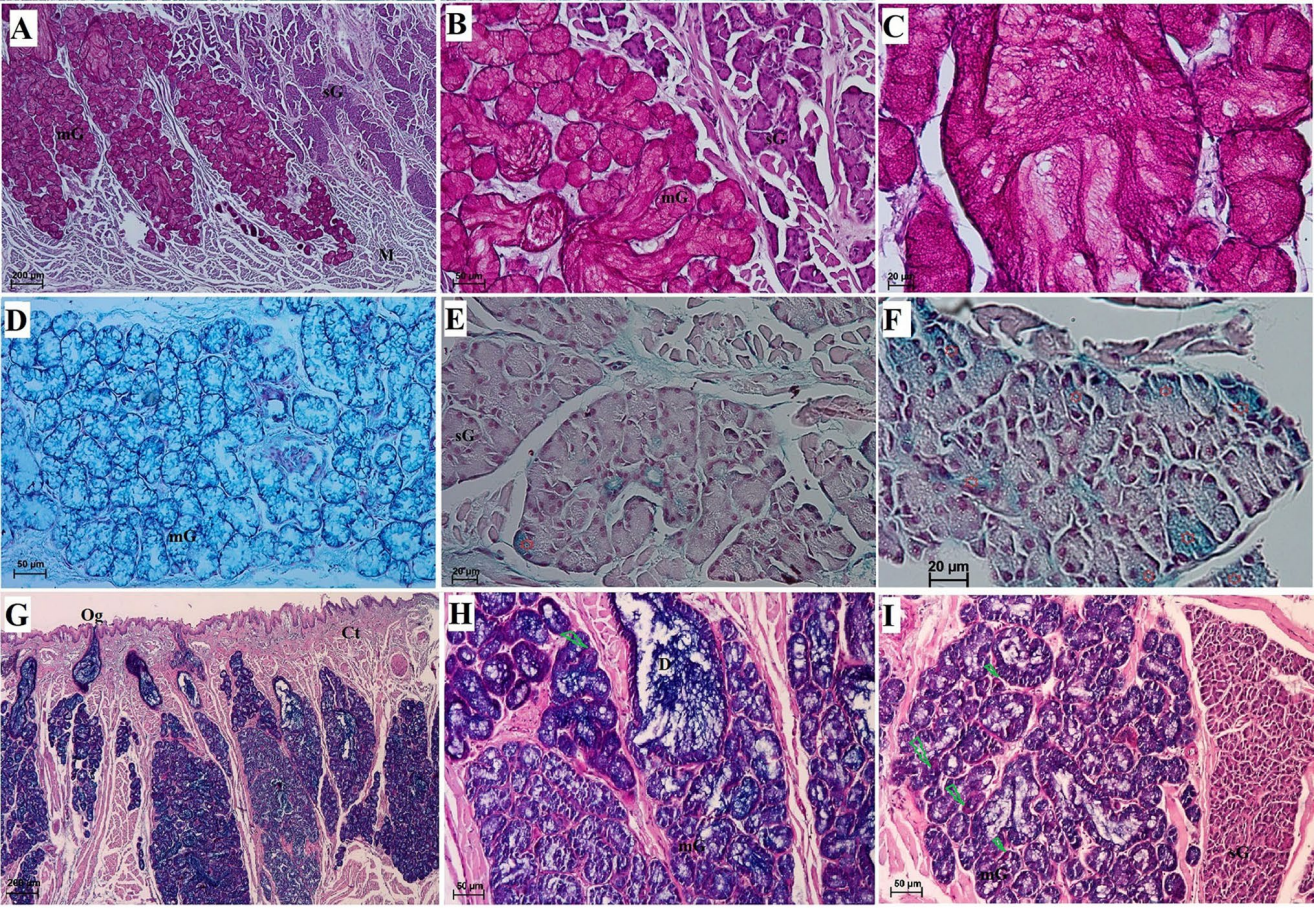
Histochemical analysis of the posterior lingual glands of the tongue of the WWCPS rat (von Ebner’s glands and Weber’s glands). **a** Strong positive reaction (+++) in the mucous cells of mucoserous glands and a negative reaction in the serous glands. PAS staining. **b** Strong positive reaction in mucous cells (+++). PAS staining. **c** Strong positive reaction (+++). PAS staining. **d** Positive reaction (++) in mucoserous glands. HDI staining. **e** Negative reaction (−) in serous glands. HDI staining. **f** Weak positive reaction (±) (*asterisks*) in some of cells of serous acini. HDI staining. **g** Strong positive reaction in mucous cells of mucoserous glands (+++). Deep blue-acidic mucins. AB/PAS staining. **h*** Deep blue* Acidic mucins,* reddish purple* neutral mucins (*green arrowhead*). AB/PAS staining. **i** Strong positive reaction in mucous cells of mucoserous glands (+++) and negative reaction (−) in serous acini.* Deep blue *Acidic mucins,* reddish purple* neutral mucins (*green arrowhead*). AB/PAS staining. *Ct* Connective tissue, *Ep* epithelium, *mG* mucoserous glands, *M* muscles, *Og* opening of the lingual glands, *sG* serous glands.* Bars* **a**, **g** 200 µm; **b**, **d**, **h**, **i** 50 µm; **c**, **e**, **f** 20 µm

The surface of the lingual root was not covered by any mechanical papillae or gustatory papillae, and the SEM study revealed that it had an irregular surface (Fig. 9). There were numerous openings of the lingual glands on the surface of the lingual root (Fig. 9a–c). In addition, there were microfolds and micropits (Fig. 9f). No anterior lingual glands were observed in the WWCPS rats. There were posterior lingual glands that included glands that accompanied gustatory papillae (von Ebner’s glands) and glands in the lingual root (Weber’s glands) classified as minor salivary glands (Figs. 10, 11). Glands accompanying vallate papilla produced a serous secretion (Figs. 7i, 8e). In the WWCPS rats, Weber’s glands were mucoserous, with a predominance of mucous cells (Fig. 10d, e, g, h). Cells forming serous demilunes were also found between mucous cells (Fig. 10c_1_). The typical ducts were absent and were replaced by tubules, with wide lumina close to the surface of the tongue (Fig. 10b, g). Myoepithelial cells were visible around individual acini of the mucoserous lingual gland (Fig. 10c_1_). At the surface of the tongue, the openings of the lingual glands were covered by a stratified squamous epithelium (Fig. 10b). PAS staining revealed a strong positive reaction (+++) in Weber’s gland cells as well as in the tubular cells (Fig. 11a–c). Those cells stained dark red in the glands producing neutral mucin (Fig. 11a–c). The AB pH 2.5 stains gave a strong positive reaction (+++) in the cells and tubules of Weber’s glands, which stained light blue indicating the presence of cells secreting acidic sulphated mucins (Fig. 10e–g). The AB pH 1.0 stains gave a positive reaction (++) in the cells of Weber’s glands and in the tubules (Fig. 10h, i) confirmed the presence of acidic carboxylated mucins. There was a negative reaction in the serous demilunes, which consisted of the secretory ducts together with cells producing mucus (Fig. 10e). HDI staining showed a (++) positive reaction in the seromucous glands, indicating the production of carboxylated mucosubstances (Fig. 11d), while serous glands showed either a negative (−) or weak positive reaction (±) in HDI staining (Fig. 11e, f). The AB/PAS staining gave a strong positive reaction (+++) in the cells and tubules of Weber’s glands and a negative reaction in serous von Ebner’s glands (Fig. 11g, h, i). Dominant positive dark blue AB/PAS mucous cells producing acidic mucins were present (Fig. 11g, h, i). On the other hand, single cells stained reddish-purple showed weak production of neutral mucins (Fig. 11g, h, i). Histochemical analysis of WWCPS lingual Weber’s glands demonstrated the presence of mixed mucoserous secretion, with dominance of mucous cells which produced acidic mucins.

### TEM study

**Fig. 12a–d:**
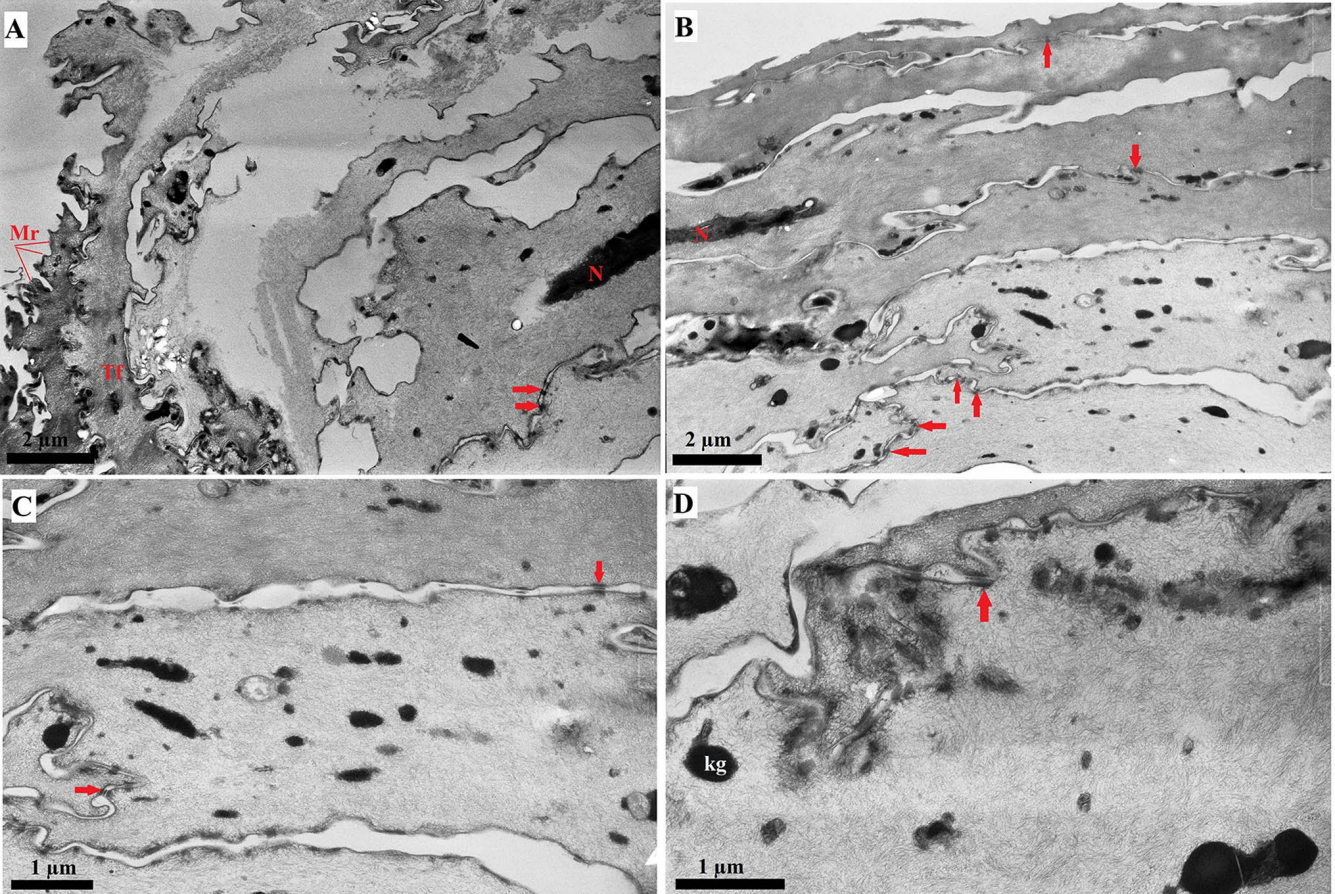
Characterisation of the dorsal lingual epithelium in the WWCPS rat tongue using transmission electron microscopy (TEM). **a**, **b** Easily visible microridges on the surface of epithelial cells. **c** Magnification of epithelial cells with numerous desmosomes. **d** Easily visible desmosomes and tonofilaments. *Red arrows *Desmosomes, *kg* keratohyaline granules, *Mr* microridges, *N* nucleus, *Tf* tonofilaments.* Bars* **a**, **b** 2 µm; **c**, **d** 1 µm

**Fig. 13a–d:**
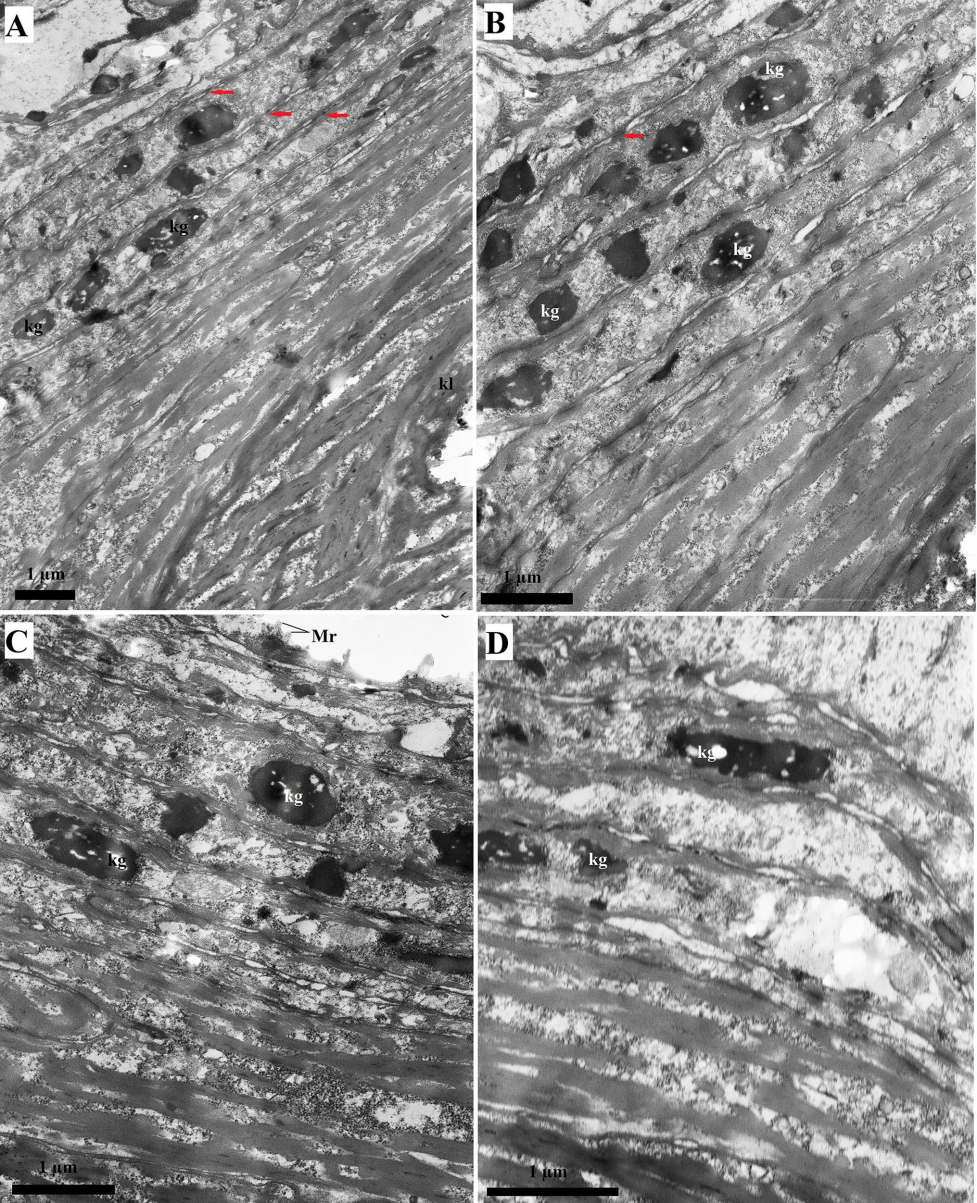
Characterisation of the keratohyaline granules in the dorsal lingual epithelium of the WWCPS rat tongue using TEM. **a** epithelial cells in deep layer of anterior part of filiform papillae with numerous keratohyaline granules, and epithelial cells of posterior side of filiform papillae with keratinised layer. **b** Magnification of keratohyaline granules and keratinised layer. **c** Elongate in shape keratohyaline granules. **d** Magnification of keratohyaline granules. *kg* Keratohyaline granules, *kl* keratinized layer, *Mr* microridges.* Bars* 1 µm

The epithelium lining the dorsal surface of the tongue contained four layers of cells: the basal, spinous, granular and keratin layers (Figs. 12, 13). The cells in the basal layer contained oval nuclei. The nuclei in the spinous layer and granular layer were more elongated (Fig. 12). Numerous intercellular connections in the form of desmosomes (Fig. 12) were present between cells, particularly in the superficial layers. Numerous microridges (Fig. 12a) were present on the surface of the cells in the superficial layer. Moreover, numerous tonofilaments (with low electron density) were observed within the epithelial cells (Fig. 12). Amorphous fragments were present in the cells of the superficial layers of the epithelium, while no other intracellular inclusions were visible (Fig. 12). Keratohyaline granules were present in the cells of the intermediate layer of the epithelium, especially in the anterior basal part of the filiform papillae, while the posterior side of these papille was covered by a keratinised surface layer (Fig. 13). Keratohyaline granules with high electron density were irregular or elongated (Fig. 13).

## Discussion

Studies that assessed the structure of the tongue in chosen mammals living in different habitats and receiving different diets have found there are species-specific morphological differences (Iwasaki and Miyata 1990; Chamorro et al. 1993a, b; Saber et al. 2001; Jackowiak 2006; Toprak and Yilmaz 2007; Elsnasharty et al. 2013; Yoshimura et al. 2008, 2013; Erdoğan et al. 2015, 2016; Goździewska-Harłajczuk et al. 2015, 2016; Akbari et al. 2017).

The tongues of the WWCPS rats had a macroscopic structure comparable to the tongues of other rat lines (Al-Refai et al. 2014), the Wistar rat (Kullaa-Mikkonen et al. 1987, Picoli et al. 2006; Verli et al. 2008; Costa et al. 2013) or the Sprague–Dawley rat (Costa et al. 2013; Reginato et al. 2014). The median groove in the WWCPS was approximately 1 cm long and was found only in the apex. A similar well demarcated median groove in the region of the apex was found in rodents such as the bank vole (Jackowiak and Godynicki 2005), porcupine (Karan et al. 2011), the squirrel (Ünsaldi 2010), agouti (Ciena et al. 2013), hazel dormouse (Wołczuk 2014), the Persian squirrel (Sadeghinezhad et al. 2018), the large bamboo rat (Wannaprasert 2018) and at the apex and body of the tongue in the degu (Cizek et al. 2016). In contrast to wild rats, the median groove was absent in the guinea pig (Kobayashi 1990), Patagonian cavy (Emura et al. 2011) and capybara (Watanabe et al. 2013). In addition, the intermolar prominence was well marked in the WWCPS rat, similarly to the Wistar and Sprague-Dawley rats (Costa et al. 2013) and other rodents, including the Patagonian cavy (Emura et al. 2011), the porcupine (Karan et al. 2011), the rabbit (Nonaka et al. 2008; Abumandour and El-Bakary 2013), hazel dormouse (Wołczuk 2014), the degu (Cizek et al. 2016) and the large bamboo rat (Wannaprasert 2018). It was poorly visible in the agouti (Ciena et al. 2013) and absent in the Persian squirrel (Sadeghinezhad et al. 2018) and capybara (Watanabe et al. 2013). The filiform papillae were the most common papillae in the WWCPS rat tongue. This was similar to the findings in other members of the Rodentia order (Whitehead and Kachele 1994; Watanabe et al. 1997; Emura et al. 1999a, b; Jackowiak and Godynicki 2005; Ünsaldi 2010; Kulawik and Godynicki 2007; Nonaka et al. 2008; Cheng et al. 2009; Kilinc et al. 2010; Atalar and Karan 2011; Karan et al. 2011; Emura et al. 2011; Abumandour and El-Bakary 2013; Ciena et al. 2013; Sakr et al. 2013; Watanabe et al. 2013; Wołczuk 2014; Cizek et al. 2016; Wannaprasert 2018; Sadeghinezhad et al. 2018). The filiform papillae in the WWCPS rats were of various sizes depending on their localisation on the surface of the tongue. Based on the SEM study, four subtypes of filiform papillae were identified in the WWCPS rat. In the Sprague Dawley and Wistar rats, three subtypes were described: simple conic papillae, giant papillae and true papillae (Costa et al. 2013; Reginato et al. 2014). In addition, Iwasaki et al. 1987 characterized conical-shaped filiform papillae, large conical papillae and filiform papillae with several long and slender branches in rats. In general, the structure of the filiform papillae in the WWCPS rats was similar to that in the Wistar or Sprague-Dawley rat (Iwasaki et al. 1997, 1999; Picoli et al. 2006). The analysis of the surface of the tongue in the WWCPS rats revealed that keratohyaline granules were present in the anterior part of the processes of filiform papillae and contained soft keratin, while the hard cortex comprising keratin did not contain keratohyaline granules in the posterior part of the filiform papillae processes. This is consistent with the findings of Kullaa-Mikkonen et al. (1987) in Wistar rats. The study of Iwasaki et al. (1999) on Sprague-Dawley rats confirmed that the morphogenesis of the filiform papillae may be influenced by various factors, affecting the shape of the anterior and posterior part of the filiform papillae. The dietary intake of wild rats is usually different to that of laboratory rats. Hence, the degree of keratinisation of the epithelial surface of the filiform papillae may vary in wild rats compared to laboratory rats. However, in this study, the wild-type WWCPS rats were fed a laboratory feed. Any differences between the animals are genetic or epigenetic and are not caused by environmental factors in ontogenesis.

In other rodents, the shape of the filiform papillae differed from those in the WWCPS rats. In the guinea pig, filiform papillae with a long central tip and several shorter lateral tips on the apex, a large conical papilla and small conical papillae containing rod-shaped projections (Kobayashi 1990; Ciena et al. 2017) were identified. In the porcupine, the filiform papillae were slender and conical or large with a round apex. Some of them were cylindrical and had a sharp apex (Karan et al. 2011). In the WWCPS rat, the giant filiform papillae located on the intermolar prominence were directed rostrally, similarly to those in the porcupine (Karan et al. 2011). The remaining types of filiform papillae in the WWCPS rats were directed caudally. Several subtypes of filiform papillae were also present in the bank vole: slim filiform papillae, larger filiform papillae, conical papillae and saw-like papillae (Jackowiak and Godynicki 2005). Large conical papillae were found in the Patagonian cavy (Emura et al. 2011) and three (Nonaka et al. 2008) to five (Abumandour and El-Bakary 2013) subtypes of filiform papillae were identified in the rabbit. Similarly, several filiform papillary subtypes were identified in the large bamboo rat (Wannaprasert 2018). As in the WWCPS rats, the closer the papillae were to the vallate papilla, the wider their base, leading to the formation of wide filiform papillae. In the degu, the filiform papillae on the apex had two spines (Cizek et al. 2016). Such spines were not identified in the WWCPS rats in the apex. Interestingly, in the degu, conical papillae were present on the root of the tongue. In the capybara, the shape of the filiform papillae (Watanabe et al. 2013) differed from that in the WWCPS rats.

As in other rodents, the gustatory papillae in the WWCPS rats could be divided into fungiform, foliate and vallate papillae. The fungiform papillae were round and dome-like, similar to those in the Sprague-Dawley rats (Reginato et al. 2014). In the Persian squirrel, the fungiform papillae contained from one to four taste buds distributed on the dorsal surface of the tongue (Sadeghinezhad et al. 2018). The surface of the fungiform papillae in the large bamboo rat also contained from one to four taste pores (Wannaprasert 2018). The surface of the fungiform papillae in the WWCPS rat contained from one to two taste pores, while in the bank vole, the fungiform papillae were round and contained a single taste pore on the surface (Jackowiak and Godynicki 2005). In the agouti, the fungiform papillae were also dome-like (Ciena et al. 2013). While in the WWCPS rats the fungiform papillae were located on the apex and body of the tongue, they were located on the entire surface of the tongue in the capybara, with the least papillae located on the lingual root (Watanabe et al. 2013). In contrast to the WWCPS rats, there were no fungiform papillae on the surface of the tongue in the degu (Cizek et al. 2016).

There was a single vallate papilla and an incomplete papillary groove in the WWCPS rat, which was also reported in the Sprague-Dawley rat (Reginato et al. 2014). The incomplete papillary groove was located in the caudal part of the vallate papilla. In addition, the annular pad was poorly developed. In the rat, the surface of the vallate papilla was covered with a transverse groove and elevations, besides a few taste pores were present on their surface (El Sharaby et al. 2014). Other rodents had from one to four vallate papillae. Three V-shaped vallate papillae were found in the Persian squirrel (Sadeghinezhad et al. 2018), two oval vallate papillae were described in the large bamboo rat (Wannaprasert, 2018) and a single vallate papilla was present in the bank vole (Jackowiak and Godynicki 2005). The vallate papillae in the WWCPS rats also differed from the one in the agouti in that it had four elongated vallate papillae (Ciena et al. 2013). Two vallate papillae in the degu were surrounded by an incompletely formed groove (Cizek et al. 2016). Two vallate papillae were described in the capybara (Watanabe et al. 2013), the Patagonian cavy (Emura et al. 2011) and the rabbit (Nonaka et al. 2008; Abumandour and El-Bakary 2013), while three papillae were identified in the squirrel (Ünsaldi 2010).

The foliate papillae in the WWCPS rat were located symmetrically on the latero-caudal part of the tongue between the body and the root. In the bank vole, the foliate papillae had a similar localisation (Jackowiak and Godynicki 2005). The foliate papillae in the WWCPS rat was formed from five pairs of epithelial folds similarly to the Sprague-Dawley rats (Reginato et al. 2014). The finger-like foliate papillae that were formed from several folds were also present in the Persian squirrel (Sadeghinezhad et al. 2018). In the agouti, the foliate papillae were formed by 12 ridges separated by grooves located on either side of the lateral and dorsal surface of the tongue in its caudal region (Ciena et al. 2013). Similarly to the WWCPS rats, well-formed foliate papillae were also described in the capybara (Watanabe et al. 2013), Patagonian cavy (Emura et al. 2011), squirrel (Ünsaldi 2010) and rabbit, where they were oval (Abumandour and El-Bakary 2013) and separated by a parallel groove (Nonaka et al. 2008). According to Kobayashi 1990, the guinea pig had two foliate papillae. In contrast, no foliate papillae were found in the large bamboo rat (Wannaprasert 2018). In the WWCPS rat, the taste buds in the walls of the foliate papillae were elongated and well-formed. Interestingly, in the mouse, the taste buds of the foliate papillae develop and function in the first 8 days of life (Toprak and Yilmaz 2007).

As in the WWCPS rats, Weber’s glands in the lingual root in the Sprague-Dawley rats were mixed tubulo-acinar glands (Nagato et al. 1997). The study by Nagato et al. 1997 did not find typical ducts in Weber’s glands in the Sprague-Dawley rat, which was comparable to the WWCPS rat lingual gland in our study. In the WWCPS, typical mucous cells were present in the walls of the lingual mucoserous glands, which has been previously shown in the Sprague-Dawley rat (Nagato et al. 1997). Moreover, the openings of the mucoserous glands, which were present at the surface of the tongue in the WWCPS rat, were covered by a stratified keratinised epithelium, as reported by Nagato et al. 1997. In the Persian squirrel, a PAS positive reaction indicated the presence of neutral mucin in those glands (Sadeghinezhad et al. 2018), as in the WWCPS rat, where the intense colour of the reaction confirmed a positive outcome. AB pH 1.0 and AB pH 2.5 in the Persian squirrel stained the mucous cells of the glands blue, indicating that they secrete acidic carboxylated mucin as well as acidic sulphated mucin (Sadeghinezhad et al. 2018), similarly to the WWCPS rat. However, in WWCPS rats, AB pH 2.5 stained the mucous cells more intensely than AB pH 1.0. In addition, the AB/PAS stains in the WWCPS rats indicated that the mucous cells producing acidic mucins were predominant, which was comparable to the Persian squirrel (Sadeghinezhad et al. 2018). On the other hand, some differences in the type of secretion produced were found between the WWCPS rat and the mole rat (Kuru et al. 2017). In the degu, the posterior lingual gland also had a mixed character and was divided into several lobules (Cizek et al. 2016). Besides similarly to WWCPS rat the serous acini of von Ebner’s glands were observed in capybara (Watanabe et al. 2013). In the hamster, von Ebner’s gland with serous acini and Weber’s glands with dominant mucous cells were observed (Cheng et al. 2009), which was comparable to the structure of the WWCPS rat lingual gland. Additionally, the shape of the serous and mucous cells of the lingual gland in both the hamster (Cheng et al. 2009) and WWCPS rat was similar. In Syrian hamsters, von Ebner’s glands have a similar histologic structure to those same glands in the rat. However, the enzymatic activity within those glands varies between the two species (Paliwal et al. 2006). The secretion in the posterior lingual glands in the WWCPS rats is similar to that of the Sprague-Dawley rat, enabling swallowing. Furthermore, von Ebner’s gland facilitates the sensation of taste by rinsing the area of the gustatory papillae (Nagato et al. 1997). The study by Youn and Jo (1998) found that in rats the composition of the secretion produced by the lingual salivary glands changes with the growth of the animal.

The study using the TEM of the lingual surface of the WWCPS rat revealed the presence of four cell layers, similarly to the agouti (Ciena et al. 2013), capybara (Watanabe et al. 2013) or Sprague–Dawley rat (Iwasaki et al. 1999). TEM of the WWCPS rat tongue also confirmed the presence of numerous keratohyaline granules in the intermediate layer of the dorsal lingual epithelium anterior to the filiform papillae. Similar findings in the Sprague-Dawley rat were described by Iwasaki et al. (1999). The shape of the keratohyaline granules in the WWCPS rats ranged from irregular to elongated, which was also been observed in other laboratory rats (Iwasaki et al. 1999). Keratohyaline granules, also observed in the granulous layer of the capybara lingual epithelium, were comparable to those in the WWCPS rat (Watanabe et al. 2013).

Rats are omnivores, and wild rats have access to diverse food. In addition, wild rats fight for food and behave differently to laboratory rats. Typical laboratory lines of rats have constant access to food, which is not diversified and usually consists of ready-made feeds. Comparative postnatal histomorphogenesis of the mandible in wild and laboratory mice showed that there are differences between wild and laboratory animals in the genetic regulation of bone remodeling (Martinez-Vargas et al. 2018). The study of Martinez-Vargas et al. (2018) found that inbred mice may be used as a basic animal study model although changes instigated by domestication need to be considered.

In conclusion, current physiological studies in wild, wild vs laboratory rats and WWCPS rats are valuable as they enable the identification of traits formed by domestication of lines of breeding rats. However the morphological analysis of the wild-type rats found that the general morphology of the tongue and microstructure, as well as the distribution of the lingual papillae are similar (based on histological, SEM and TEM studies) to the Wistar or Sprague-Dawley laboratory rats. Despite the previously described similarities between strains of laboratory rats and the WWCPS rat, the *SEM* study revealed the presence of four distinct subtypes of filiform papillae in the WWCPS rat.
